# Geometric and topological approaches to shape variation in *Ginkgo* leaves

**DOI:** 10.1098/rsos.210978

**Published:** 2021-11-24

**Authors:** Haibin Hang, Martin Bauer, Washington Mio, Luke Mander

**Affiliations:** ^1^ Department of Mathematical Sciences, University of Delaware, Newark, DE 19716, USA; ^2^ Department of Mathematics, Florida State University, Tallahassee, FL 32306, USA; ^3^ School of Environment, Earth and Ecosystem Sciences, The Open University, Milton Keynes, MK7 6AA, UK

**Keywords:** biological shape, plant traits, morphometric methods, elastic curves, persistent homology

## Abstract

Leaf shape is a key plant trait that varies enormously. The range of applications for data on this trait requires frequent methodological development so that researchers have an up-to-date toolkit with which to quantify leaf shape. We generated a dataset of 468 leaves produced by *Ginkgo biloba*, and 24 fossil leaves produced by evolutionary relatives of extant *Ginkgo*. We quantified the shape of each leaf by developing a geometric method based on elastic curves and a topological method based on persistent homology. Our geometric method indicates that shape variation in modern leaves is dominated by leaf size, furrow depth and the angle of the two lobes at the leaf base that is also related to leaf width. Our topological method indicates that shape variation in modern leaves is dominated by leaf size and furrow depth. We have applied both methods to modern and fossil material: the methods are complementary, identifying similar primary patterns of variation, but also revealing different aspects of morphological variation. Our topological approach distinguishes long-shoot leaves from short-shoot leaves, both methods indicate that leaf shape influences or is at least related to leaf area, and both could be applied in palaeoclimatic and evolutionary studies of leaf shape.

## 1. Introduction

Leaf shape is a fascinatingly diverse plant trait. It can vary between taxa, between individuals in different populations of the same species, and for some species there are striking variations in leaf shape within a single plant, a phenomenon known as heterophylly. Additionally, different regions of a leaf expand at different rates during development, and this leads to allometric changes in shape as a leaf grows. Leaves are primary sites of photosynthesis and play a central role in the growth and survival of a plant, and for flowering plants work has shown that variation in leaf shape may be related to thermoregulation [1], the constraints of hydraulics [2] and biomechanics [3], patterns of development and leaf expansion [4], as well as the avoidance of herbivory [5] and the optimal interception of light (see [6] for a review). Leaf shape is, therefore, a trait for which there are many functional trade-offs, and from an ecological perspective may be viewed ‘not as a single major axis, but rather as an option that fine tunes the leaf to its conditions over both short and evolutionary time spans' [6, p. 547].

The taxonomic and ecological significance of leaf shape has led to the development of numerous methods to characterize this trait. Certain methods rely on largely qualitative observation. For example, aspects of leaf shape can be described using specialist terminology [7], which allows leaves to be placed into categories based on their gross morphology, and this approach has proved useful in studies of plant architecture (e.g. [8,9]) and studies of fossil leaves that may not be preserved in their entirety (e.g. [10]). Other methods for characterizing leaf shape are based on morphometric measurements of certain features on a leaf, which can either be made manually by human researchers or computationally using image analysis software. For example, Leigh *et al*. [11] described leaf shape using measurements of leaf area and leaf dissection (leaf perimeter/area) in the context of plant hydraulics, and Royer *et al*. [12] used the same measure of leaf dissection to investigate the relationship between mean annual temperature and leaf shape. Measurements of such morphological features are often used to generate indices of leaf shape, such as compactness (perimeter^2^/area) and shape factor (4π × leaf area/perimeter^2^), which are used to summarize aspects of leaf shape and show how it relates to the environment or has changed through time [13–16]. Additionally, Shi *et al*. [17] found that the leaf shape of bamboo could be depicted by the simplified Gielis equation, while Li *et al*. [18] noted that leaf shape of two *Michelia* species followed the superellipse equation, and Shi *et al*. [19] developed a general formula for describing ovate leaf shape in plants. Morphometric techniques that use landmarks (a constellation of discrete anatomical loci, each described by two- or three-dimensional Cartesian coordinates to quantify morphology [20–22]) have been employed to capture variation in leaf shape [23] and have highlighted differing developmental and evolutionary contributions to leaf shape [24], while elliptic Fourier analysis has been used to quantify leaf outlines [25,26]. Persistent homology (PH)—a topological data analysis method—has also been applied to the problem of quantifying leaf shape [27,28], and represents a morphometric framework to measure plant form that allows comparison of the morphology of different plant organs such as leaves, roots and stems [29,30].

Owing to the diversity of leaf form—and the range of applications for data on leaf morphology—regular methodological experimentation is required so that researchers have an up-to-date toolkit with which to quantify this plant trait. In this paper, we provide such experimentation through a quantitative study of leaf shape in *Ginkgo biloba* L., an extant gymnosperm. We have selected *Ginkgo* as a study system primarily because of the diversity of leaf shapes that are produced by individual specimens (e.g. [11]) and because of the palaeobotanical importance of fossil *Ginkgo* and its extinct evolutionary relatives. In particular, *Ginkgo* and its relatives were important elements of Earth's vegetation during the Mesozoic Era (approx. 250–65 Ma) and fossil leaves of plants that are evolutionary ancestors of living *Ginkgo* are commonly found in sedimentary rocks. These leaves have been widely used to investigate Earth's ancient atmospheres and environments using their stomatal indices, carbon isotopic composition and physiognomy (see [16,31,32]). Consequently, with a view to demonstrating the applicability of our methods to fossil material our study includes a small number (24) of fossil *Gingko* leaves.

Previous work on *Ginkgo* leaf morphology has shown that this plant is characterized by pronounced heterophylly with different leaf forms borne on long shoots versus short shoots [33,34]. The leaves of long shoots are typically smaller and can have a deep wide furrow and a dissected margin, while the leaves of short shoots are typically larger and can have a less pronounced furrow [11]. The variability of *Ginkgo* leaf morphology is emphasized by measures of specific leaf area (the ratio of leaf lamina area to leaf lamina dry mass), which indicate that the form of *Ginkgo* leaves varies not only between the long and short shoots of the plant, but also between the trees of different genders (micro- versus megasporangiate), as well as between juvenile and mature portions of a megasporangiate canopy, and also for short shoots bearing seed and adjacent short shoots without seed ([35], see also [36]). The hydraulic architecture of *Ginkgo* leaves has been quantified [37] and it has been shown that long-shoot and short-shoot *Ginkgo* leaves have different structural and hydraulic properties, probably related to the greater hydraulic limitation of long-shoot leaves during leaf expansion [11]. Aspects of *Ginkgo* leaf shape are demonstrably sensitive to atmospheric composition, and when quantified by shape factor, extant *Ginkgo* leaves that have been subject to elevated atmospheric SO_2_ levels in controlled environment chambers are significantly rounder than control leaves [16]. Additionally, *Ginkgo* leaf shape may also be sensitive to elevation, although the positive relationship between the length : width ratio of leaves and elevation is weak [38].

Our study builds on this body of previous work by taking an exploratory approach to the morphology of *Ginkgo* leaves. We do not initially focus on any specific morphological features such as leaf length or the nature of the leaf margin, but instead use geometric and topological methods to reveal the features that explain the observed variation in leaf shape. Our overall goal is to provide an illustration of how these methods can be applied to the problem of quantifying leaf shape, and our specific aims are as follows: (i) to develop a geometric method and a topological method for quantifying leaf shape; (ii) to apply these methods to the leaves of living *Ginkgo* in order to reveal which features explain the observed variation in the shape of sampled leaves; (iii) to compare the results produced by the two methods in order to explore the degree to which they reveal different aspects of morphological variation; and (iv) to apply our methods to fossil leaves of ancient evolutionary relatives of living *Ginkgo* in order to confront a degree of morphological variation not present in our sample of living *Ginkgo*, and to demonstrate how they could be used to study the evolution of leaf shape through geological time.

## 2. A dataset of modern and fossil leaves

Mature and fully expanded leaves were harvested from a reproductively immature *G. biloba* tree growing in partial shade as an introduced specimen on the campus of The Open University, UK. The specimen measures 161 mm at breast height and was ascended using a ladder. Seven branches growing towards the west at approximately halfway up the specimen were removed from the trunk using a saw. Every leaf growing on each branch was plucked from the base of the petiole and dried in a plant press. A total of 468 leaves from a mixture of short shoots and long shoots were collected from the specimen. Each of these leaves was photographed next to a scale bar using a digital camera positioned 20 cm above a light box. Twenty-two fossil leaves produced by evolutionary relatives of living *G. biloba* were extracted from the collections of the Natural History Museum in London, and two fossil leaves were extracted from the geology collections of the School of Environment, Earth and Ecosystem Sciences, The Open University (table 1). Each fossil leaf was photographed next to a scale bar using a digital camera and the outline of each fossil was traced using Adobe Illustrator to create a digital outline of each leaf. The petioles of fossil leaves are frequently broken, distorted or completely absent as a result of the fossilization process. A central goal of our manuscript is to compare living and fossil *Ginkgo* leaves and in order to facilitate this, we have excluded the petiole from our analyses. Our analyses are, therefore, focused on the shape of *Ginkgo* leaf blades. Our dataset of modern and fossil *Ginkgo* leaf images is available online (see Data accessibility).

**Table 1.** Fossil *Ginkgo* leaves housed in the collections of the Natural History Museum, London, and The Open University that we have investigated in this paper.

specimen name	accession number	age	country	location	specimen number (this study)
*Ginkgo cranei*	NHM: V.68763	Palaeocene	United States	North Dakota	fossil_1
*Ginkgo cranei*	NHM: V.68764	Palaeocene	United States	North Dakota	fossil_2
*Ginkgo gardneri*	NHM: V.14834	Eocene	Scotland	Isle of Mull	fossil_3
*Ginkgo gardneri*	NHM: V.14838	Palaeocene/Eocene	Scotland	Isle of Mull	fossil_4
*Ginkgo gardneri*	NHM: V.18436	Eocene	Scotland	Isle of Mull	fossil_5
*Ginkgo gardneri*	NHM: V.24999	Eocene	Scotland	Isle of Mull	fossil_6
*Ginkgo sp.*	NHM: V.2477	Eocene	Scotland	Isle of Mull	fossil_7
*Ginkgo digitata*	NHM: V.24587	Cretaceous	Australia	Queensland	fossil_8
*Ginkgo digitata*	NHM: V.39211	Jurassic	England	Yorkshire	fossil_9
*Ginkgo digitata*	NHM: V.13503	Jurassic	England	Yorkshire	fossil_10
*Ginkgo digitata*	NHM: V.10316	Jurassic	England	Yorkshire	fossil_11
*Ginkgo huttonii*	NHM: V.60195	Jurassic	England	Yorkshire	fossil_12
*Ginkgo huttonii*	NHM: V.3580	Jurassic	England	Yorkshire	fossil_13
*Ginkgo huttonii*	NHM: V.40511	Jurassic	England	Yorkshire	fossil_14
*Ginkgo huttonii*	NHM: V.39210	Jurassic	England	Yorkshire	fossil_15
*Ginkgo huttonii*	NHM: V.978	Jurassic	England	Yorkshire	fossil_16
*Ginkgo huttonii*	NHM: V.979	Jurassic	England	Yorkshire	fossil_17
*Ginkgo longifolius*	NHM: V.39209	Jurassic	England	Yorkshire	fossil_18
*Ginkgo siberica*	NHM: V.58618	Jurassic	England	Yorkshire	fossil_19
*Ginkgo digitata*	NHM: V.3423	Jurassic	England	Gloucestershire	fossil_20
*Ginkgo digitata*	NHM: V.3429	Jurassic	England	Gloucestershire	fossil_21
*Ginkgo siberica*	NHM: V.19238	Jurassic	Russia	Irkutsk	fossil_22
*Ginkgo huttonii*	Open University geology collection	Jurassic	England	Yorkshire	fossil_23
*Ginkgo huttonii*	Open University geology collection	Jurassic	England	Yorkshire	fossil_24

## 3. A geometric approach to quantifying the shape of leaves

### 3.1. Methods

**Figure 1.**
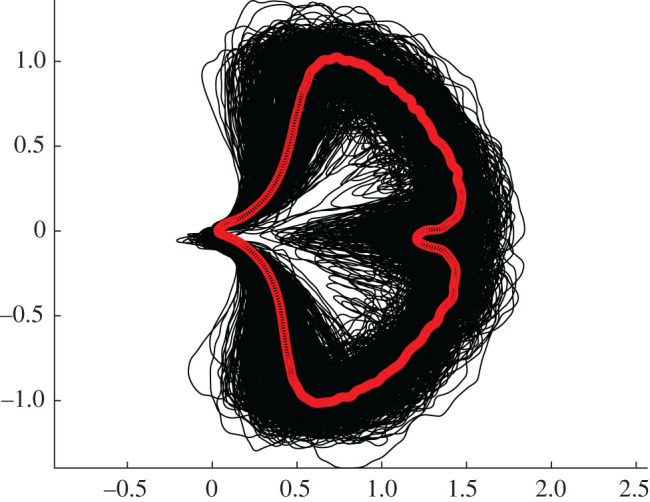
Collection of all 468 *Ginkgo biloba* leaves in our dataset represented by their boundary curves (black lines) with the Karcher mean leaf shape superimposed (red line).

Building on previous work using elastic curves to quantify leaf shape [39,40], we represented each *Ginkgo* leaf blade by its boundary curve, with values mapped in the plane (two-dimensional Euclidean space) (figure 1). When considering these representations of *Ginkgo* leaves we factored out the actions of rotation and translation and reparametrization. For example, two identical leaves could each be represented by their boundary curves, but each curve could be considered distinct from one another if they differed only by rotation (a curve could be presented at 90° on top of the other for instance), but our analysis factors out such actions. It is possible to also factor out the action of scaling and we do this in an analysis of leaf shape versus leaf area.

To quantitatively model morphological variation in our sample of *Ginkgo* leaves, we introduce a similarity measure for shapes that serves as the basis of statistical analysis. This is an intricate process for two main reasons: (i) the infinite dimensionality of the ensemble of all shapes; and (ii) the nonlinearity of shape space. To overcome this difficulty, we appeal to the concepts of Riemannian geometry, and use a Riemannian metric that quantifies the difficulty of morphing one boundary curve onto another by measuring the geodesic distance between the curves, accounting for rotations, translations and reparametrizations. This enables us to quantify shape similarity as the minimal deformation cost to reshape a curve, in this case, a *Ginkgo* leaf contour. Despite the nonlinear nature of shape space, this framework allows us to calculate mean shapes and locally linearize shape data about the mean, which, in turn, lets us employ standard statistical methods on linearized data to analyse the shape variation present in our sample of *Ginkgo* leaves.

The Riemannian metric we employ is grounded on principles of linear elasticity and is formally defined on the ensemble of parametric curves, but its invariance properties ensure that it descends to a shape metric. A precise definition of the metric and a discussion of its main properties may be found in Bauer *et al*. [41,42] (see also Klassen *et al*. [43]) for related shape metrics). In practice, the comparison of *Ginkgo* leaf boundary curves is a shape-matching problem, and to solve this we discretized the boundary curve of each leaf using a finite-dimensional representation. This reduces the problem of comparing leaf boundary curves to a finite-dimensional optimization problem that can be solved with standard methods of numerical optimization. We use principal component analysis (PCA) to uncover the principal modes of shape variation in *Ginkgo* leaves.

The essential steps in this approach are: (i) image processing to isolate each leaf from the image background and remove the petiole; (ii) find the boundary curve of each leaf blade; (iii) discretize the boundary curve of each leaf blade using a finite-dimensional representation; (iv) for each leaf blade, calculate an elastic metric that quantifies the difficulty of morphing one leaf boundary curve onto another; and (v) compare leaves and visualize the dominant modes of shape variation among leaves using PCA. The code underlying our geometric approach is fully open source and available at https://github.com/h2metrics/h2metrics. The provided package contains detailed documentation and user-friendly working examples.

### 3.2. Results

**Figure 2.**
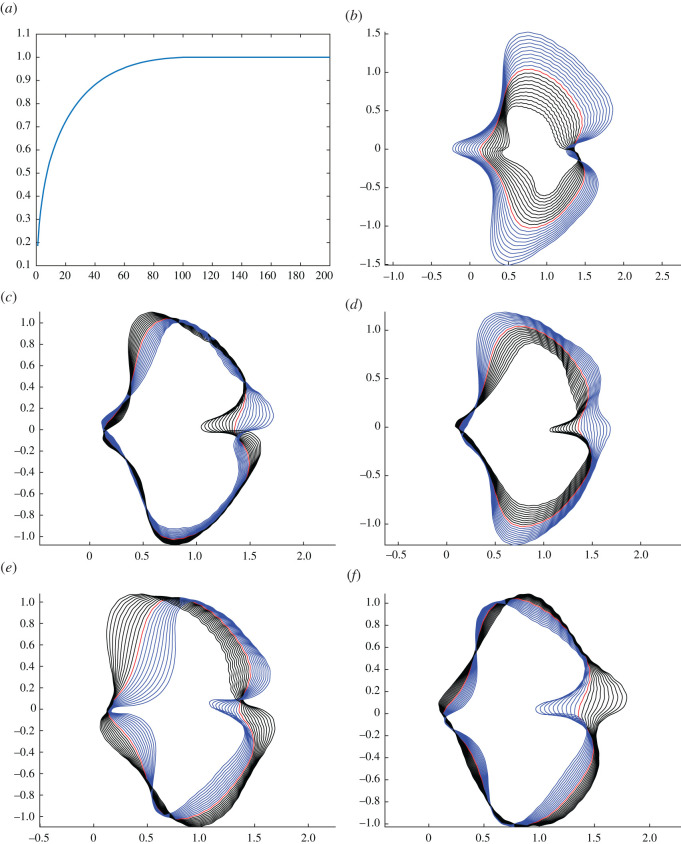
Geodesic PCA plots of *Ginkgo* leaves represented in the tangent space of the mean. Variance explained by all components (*a*), the first principle component (*b*), second principle component (*c*) and third principle component (*d*). Analysis with scaling factored out, first principle component (*e*) and second principle component (*f*).

We calculated the Karcher mean of our sample of modern *Ginkgo* leaves (figure 1) and then locally linearized the data about the mean in order to uncover the principal modes of leaf shape variation. This was accomplished by solving a shape-matching problem between the mean and each leaf in the dataset. PCA on the linearized data indicated that approximately 30 components are needed to explain 80% of the shape variation in our sample of *Ginkgo* leaves (figure 2*a*), and we graphically display the principal modes of leaf shape variation using geodesic PCA plots (figure 2*b–d*). The first mode is predominantly leaf size (first principal component, figure 2*b*), the second mode relates to the nature of the leaf margin (second principal component, figure 2*c*), and the third and fourth modes are the depth of the furrow that separates the two lobes of the typical *Ginkgo* leaf, together with the angle of the two lobes at the base of the leaf that is also related to leaf width (third principal component, figure 2*d*). Some leaves, for example, have a very deep furrow whereas others have no furrow at all. Similarly, some leaves have lobes that are quite pointed and curve backwards towards the leaf base, whereas others have lobes that do not curve backwards.

**Figure 3.**
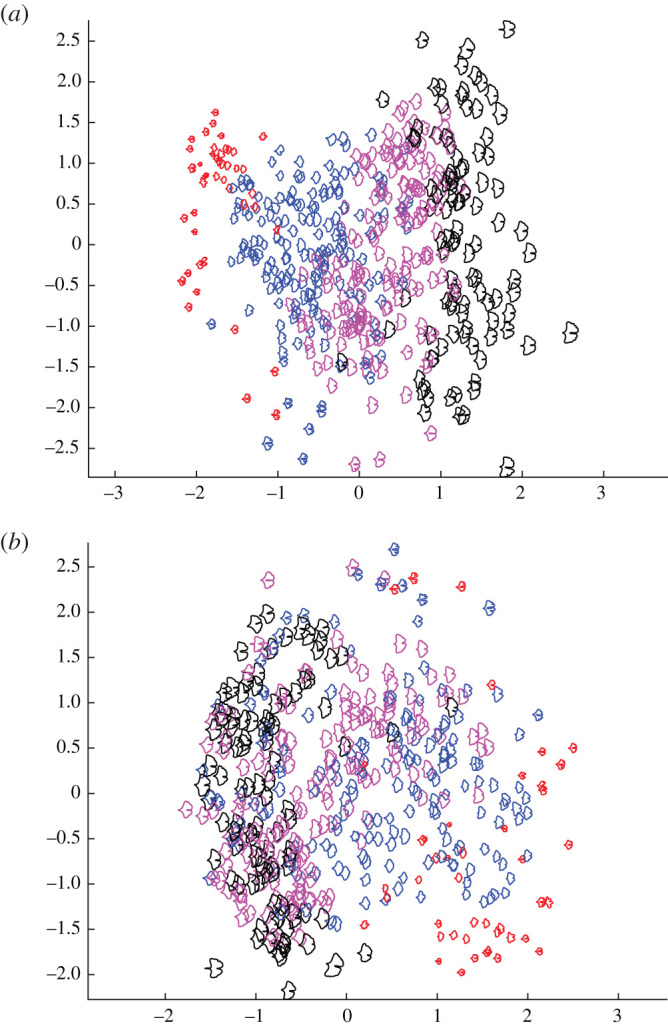
PCA ordination scatterplot (PC1 on horizontal axis, PC2 on vertical axis) showing the morphological variation among 468 modern *Ginkgo* leaves that is revealed by our geometric approach to leaf shape incorporating scale (*a*), and with scale factored out (*b*). Leaf area groups based on their areas: area ≤ 8 (red); 8 < area ≤ 16 (blue); 16 < area ≤ 24 (purple), area ≥ 24 (black); area in cm^2^.

Examples of variability in terms of the morphological features identified by our geodesic plots (figure 2*b*–*d*) can be seen in a PCA ordination of our dataset of *Ginkgo* leaves (figure 3*a*). Leaves towards the left are relatively small and leaves towards the right are relatively large (figure 3*a*). Leaves to the bottom are typically more dissected and have a relatively deep furrow, whereas leaves to the top are typically less dissected and have a relatively shallow furrow (figure 3*a*). This plot also highlights that the morphological space occupied by our sample of *Ginkgo* leaves, as delineated by our geometric approach, is organized as a continuous distribution of data points without separate clusters. Most data points are concentrated towards the centre of the ordination, and the distribution of data points becomes sparser with increasing distance from the centre (figure 3*a*).

## 4. A topological approach to quantifying the shape of leaves

### 4.1. Methods

**Figure 4.**
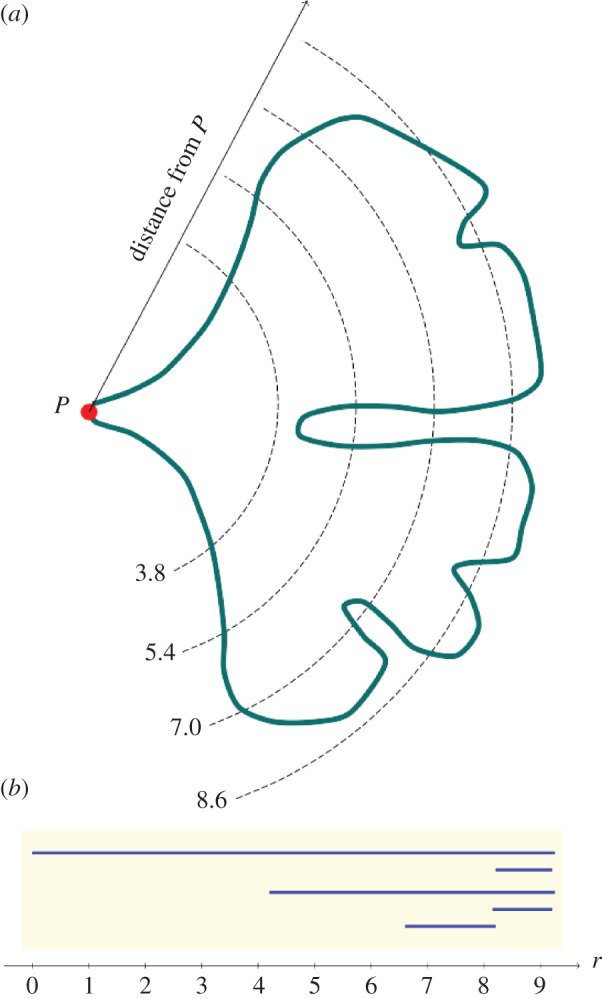
Schematic example showing the construction of a persistence barcode that describes the shape of a *Ginkgo* leaf. Four distances from the point *P* where the leaf blade meets the petiole are shown: *r* = 8.6, 7.0, 5.4, 3.8 (*a*). At the distance *r* *=* 8.6, there are four connected components outside the dashed line (*a*). At the distance *r* = 7.0, there are three connected components, at *r* = 5.4 there are two (the two lobes of the typical *Ginkgo* leaf), while at *r* = 3.8 there is one uninterrupted segment of the leaf blade contour outside the dashed line (*a*). To construct a barcode that represents a leaf, we do not count the number of connected components at widely spaced intervals as shown in (*a*). Instead, we perform a count for each *r* > 0, and record the number of connected components as *r* is gradually lowered in a barcode (*b*).

We employed the topological data analysis technique PH [27,28,44,45] and represented each *Ginkgo* leaf in our dataset with a persistence barcode. To construct this barcode, for each point on the contour of a leaf (its boundary curve), we calculated the distance to the point *P* where the leaf blade meets the petiole (figure 4*a*). The distance was measured in pixels and in our source images 152 pixels = 1 cm. All images were downscaled by 1/8 and so 19 pixels = 1 cm in our analyses. For each *r* > 0, we counted the number of connected components formed by the points on the contour whose distance to *P* is greater or equal to *r* and recorded this count as a barcode. For example, for *r* = 8.6, there are 4 connected components (these are the uninterrupted segments of the leaf blade contour, figure 4*a*), so there are *b* = 4 bars over that value of *r* (figure 4*b*). Similarly, for *r* = 7.0, 5.4, 3.8, (figure 4*a*) the corresponding number of bars is *b* = 3, 2, 1 (figure 4*b*). The barcode summarizes the count as we gradually lower the threshold *r*, with bars disappearing as connected components coalesce and bars appearing as new components emerge. The coalescence of two connected components follows the elder rule: the first-born bar survives while the younger bar dies. Through this construct, we mapped the dataset of leaves to a dataset of barcodes, with each leaf described by a barcode. In order to facilitate statistical analysis, we vectorized each barcode by listing the length of the bars in decreasing order. Since different leaves may produce barcodes with different number of bars, we padded the tails of the vectors with zeros to make all vectors the same length. In our analysis of modern leaves, statistical analyses were performed on these padded vectors. In our analysis of modern and fossil *Ginkgo* leaves combined, statistical analyses were performed on vectors that were normalized by the length of the first bar (the first component of each normalized vector was, therefore, 1 and discarded). The essential steps in this approach are: (i) image processing to isolate each leaf from the image background and remove the petiole; (ii) find the contour curve of each leaf blade; (iii) reparametrize the contour curve of each leaf blade; (iv) calculate the PH of each leaf blade; (v) construct the persistence barcode of each leaf blade; and (vi) compare leaves and visualize the dominant modes of shape variation among leaves using multi-dimensional scaling. An example of the code underlying our topological approach is available at https://github.com/haibinhang/TDA-of-ginkgo-leaves. The provided code contains documentation and user-friendly working examples.

### 4.2. Results

**Figure 5.**
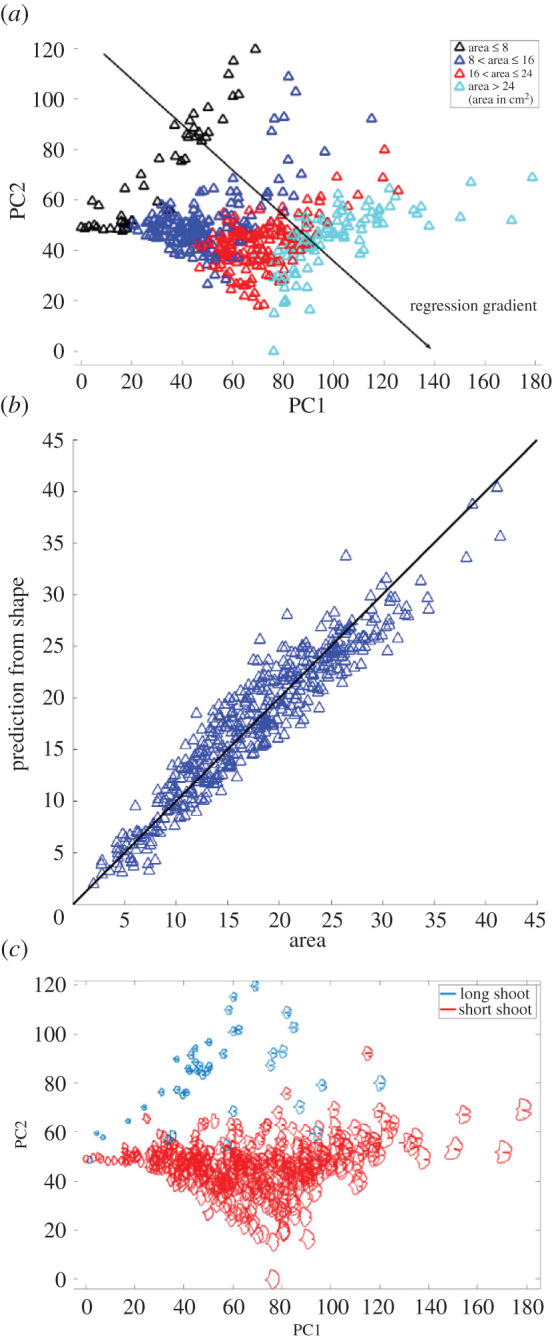
PCA ordination scatterplot showing the morphological variation among 468 modern *Ginkgo* leaves that is revealed by our topological (PH) approach to leaf shape, with data points coloured according to four leaf-area groups (*a*). Comparison of leaf area predicted from the first two principal components and measured leaf area; the vertical offset between data points and the solid black diagonal 1 : 1 line of equality indicates the discrepancy between the predicted and measured areas (*b*). PCA ordination scatterplot with long-shoot and short-shoot *Ginkgo* leaves highlighted (*c*).

Figure 5 shows the results of PCA applied to the vectorized barcode data. The first PC explains approximately 75% of the total variance and inspection of the PC loadings indicates that it is dominated by leaf length, followed by furrow depth. The second PC explains about 22% of the total variance mainly as variation in the depth of the furrow, followed by (negative) variation in leaf length. This ordination indicates that the morphological space occupied by our sample of *Ginkgo* leaves, as delineated by our topological approach, is organized as a continuous distribution of data points without separate clusters, although the majority of leaves lie in the quadrant of PC1 scores –20 to 20 and PC2 scores 0 to –20, and the leaves with PC1 scores less than 0 and PC2 scores greater than 15 are perhaps separated from the other leaves in our sample (figure 5). The two PCs show contrasting behaviour: PC1 captures a pattern in which larger leaves have a deeper furrow, whereas PC2 captures a pattern in which smaller leaves have a deeper furrow.

## 5. Application to fossil *Ginkgo* leaves

**Figure 6.**
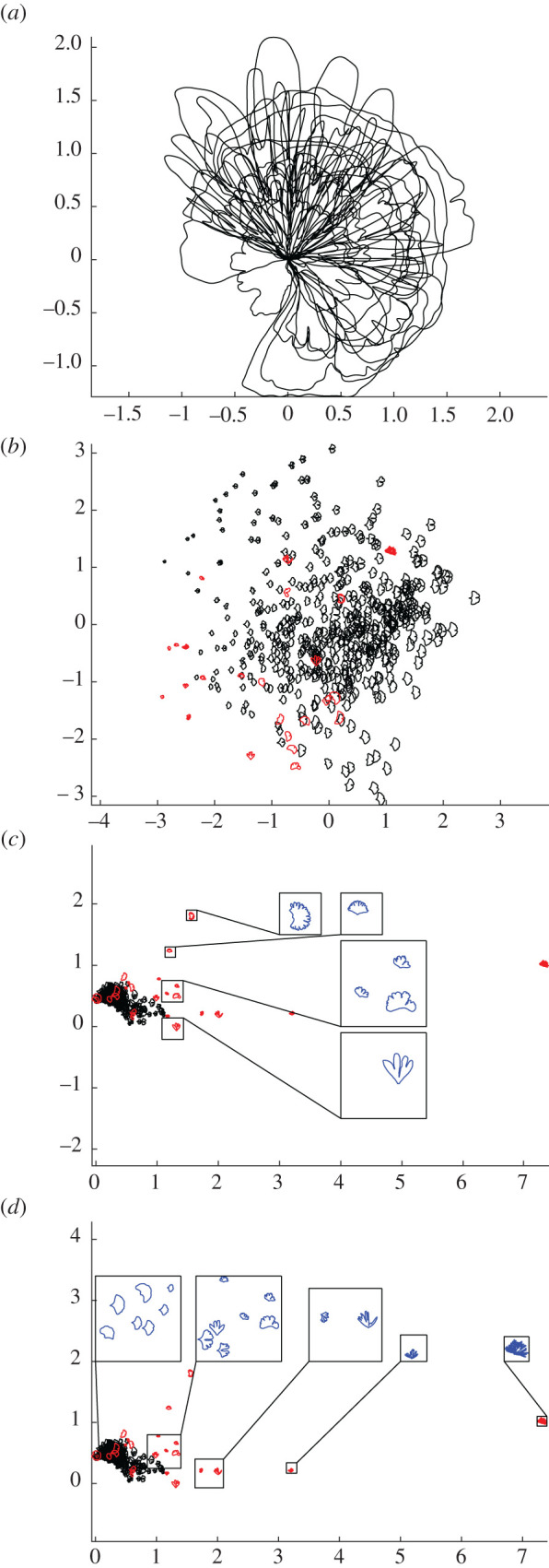
Collection of 24 fossil *Ginkgo* leaves, each represented by their boundary curves (*a*). PCA ordination scatterplot (PC1 on horizontal axis, PC2 on vertical axis) showing morphological variation of modern *Ginkgo* leaves (black data points) together with fossil *Ginkgo* leaves (red data points) based on our geometric approach, the PCs together explain 64% of the variation (*b*). Ordination showing morphological variation of modern *Ginkgo* leaves together with fossil *Ginkgo* leaves based on our topological approach, with a vertical transect of enlarged leaves highlighted in blue (*c*) and a horizontal transect of enlarged leaves highlighted in blue (*d*). Modern leaves displayed with black data points and fossil leaves displayed with red data points (*b*–*d*).

Visual inspection of fossil leaf boundary curves highlights that the diversity of leaf shapes in our collection of *Ginkgo* fossils is greater than that found in our sample of modern *Ginkgo* leaves (compare figures 1 and 6*a*). In particular, several fossil leaves are characterized by multiple deep furrows so that leaf blades consist of multiple lobes rather than just two as in the typical *G. biloba* leaf, while other fossils have highly dissected leaf margins. This greater diversity in fossil leaf shapes is picked up by both the geometric and the topological approaches we have described, and both indicate that there are fossil leaves situated outside the total range of morphological space occupied by modern *Ginkgo* leaves (figure 6*b*,*c*). Both approaches also highlight that there are some fossils leaves that are very similar to modern *Ginkgo* leaves, and there are some fossil and modern leaves that overlap in morphological space (figure 6*b*,*c*).

However, there are differences in the degree to which modern and fossil leaves are separated in morphological space using our two approaches. Using our geometric approach, relatively small leaves with shapes characterized by multiple lobes lie outside the morphological space occupied by modern *Ginkgo* leaves, while relatively large leaves with highly dissected margins plot within the space occupied by modern leaves (figure 6*b*). By contrast, using our topological approach, both of these types of fossil leaves plot outside the morphological space occupied by modern *Ginkgo* leaves (figure 6*c*). Our topological approach very clearly captures similarities and differences between modern and fossil leaves that are expected on the basis of their visual appearance alone (figure 6*c*), whereas using our geometric approach the distinction between modern and fossil leaves is not as clear (figure 6*b*).

## 6. Discussion

### 6.1. Comparison of approaches

The two approaches we have described in this paper measure leaf shape in different ways: our geometric approach is based on analysing boundary curves with an elastic metric (figure 2), whereas our topological approach is based on measuring the number of connected components as a leaf is partitioned into different segments (figure 4). Despite these differences, the two approaches both indicate that leaf size and the nature of the furrow separating the two lobes of a typical *Ginkgo* leaf are primary features that explain the observed variation in leaf shape, and both approaches also distinguish the leaves of *Ginkgo* long shoots from those of short shoots. In the PCA summary of our geometric approach, the long-shoot leaves are situated to the top left of the plot with low PC1 scores and high PC2 scores, and form a sparsely occupied region of morphological space (figure 3*a*). In the PCA summary of our topological approach, the long-shoot leaves are situated in the top left of the plot with low PC1 scores and high PC2 scores, and form a sparsely occupied region of *Ginkgo* leaf morphospace (figure 5*c*).

There are also certain differences in the morphological features pinpointed by each approach. For example, our geometric approach suggests that the angle of the two lobes at the base of the leaf (also related to leaf width) is an important mode of morphological variation in the population of leaves we have studied (figure 2*c*), but this aspect of leaf morphology is not clearly picked up by our topological approach (figure 5). Additionally, our topological approach is able to quantify the nature of the indentations in the leaf margin more clearly than our geometric approach. This is because our topological features, by design, precisely measure the depth of indentations—from large furrows to minor crenulations—in the leaf margin. The vectors we used in our topological analysis of modern and fossil *Ginkgo* leaves were normalized by the length of the first bar, and each vector, therefore, encodes the depths of the various indentations in the leaf margin relative to absolute leaf size ordered from deep to shallow. This is highlighted in the horizontal transect in figure 6*d*: to the left are modern and fossil *Ginkgo* leaves that lack indentations, whereas to the right are leaves with increasingly complex indentations, but the size of each leaf in each highlighted group varies considerably. In the language of descriptive botany, the PCA axes highlight types of leaf dissection, with axis one representing a gradient from no dissection (low axis one scores) to many relatively deep indentations (high axis one score) (figure 6*d*), and axis two representing a gradient from few relatively deep indentations (low axis two scores) to many relatively shallow indentations (high axis two scores) (figure 6*c*). This morphological feature may only be recorded in the higher orders of variation in our geometric approach (fourth and fifth principal components for our modern *Ginkgo* leaves, see figure 2*e*,*f*). The two approaches we have described are, therefore, complementary, identifying similar primary patterns of variation, but also revealing some different aspects of morphological variation.

From the perspective of PH applied to the problem of quantifying leaf shape, previous approaches have been based on measurements of the Euler characteristic curve [27,28]. Our approach is different in that we have constructed a persistence barcode from a count of connected components formed by points on a contour at incremental distances from the base of a leaf blade (figure 4), and this demonstrates an alternative means by which PH can quantify leaf shape. Often, a challenge in the use of PH is the interpretation of a persistence barcode (e.g. [45]), but for the barcodes we have generated here, the length of the longest bar represents the largest distance to *P* (figure 4) and is, therefore, a quantifier of leaf size, while the next longest bar relates to the depth of the furrow in a *Ginkgo* leaf that displays this trait, and other smaller bars relate to the depth of smaller indentations in the leaf margin. The statistical interpretation of persistence barcodes is also challenging, and as noted by Otter *et al*. [45], p. 3) for example, ‘the space of barcodes lacks geometric properties that would make it easy to define basic concepts such as mean, median, and so on’. By contrast, the framework of our geometric approach allows for the calculation of mean shapes and the linearization of data around the mean, and this highlights the complementary nature of the two approaches to leaf shape we have described in this paper.

### 6.2. Leaf shape versus leaf area: the physiognomy of *Ginkgo* leaves

Variation in leaf area is first-order mode of variation in our dataset of *Ginkgo* leaves, and experimental work on extant *Ginkgo* leaves has shown a coordinated area–shape response to elevated atmospheric SO_2_ levels. In particular, leaves grown in a high SO_2_ atmosphere were both rounder and smaller than control leaves [16]. To investigate the relationship between the area and shape of *Ginkgo* leaves in more detail we measured the area of each leaf blade using line integrals and then partitioned the leaves into four groups based on their areas (area ≤ 8, 8 < area ≤ 16, 16 < area ≤ 24, area ≥ 24; area in cm^2^). We then displayed how leaf area varies across morphological space by colour coding each of these four groups in our PCA ordinations. The colour coding highlights that for our geometric approach variation in leaf area occurs primarily along the first principal component so that leaves increase in area as their size increases (figure 3*a*). In our topological approach variation in leaf area occurs along both the first and second principal components—the boundaries between leaf area groups are orientated obliquely—indicating that leaves increase in area as their size increases and their furrows get shallower (figure 5*a*).

For our geometric approach, we investigated the relationship between leaf area and leaf shape further by undertaking an analysis of *Ginkgo* leaf blade shape in which we factored out the effects of scaling in the comparison of leaf boundary curves (two leaves were not considered distinct if they only differed in their area). Geodesic PCA plots show the first two principal components of our scale-invariant analysis, and show that the first principal component relates primarily to the shape of the leaf blade at its base (figure 2*e*) and the second principal component relates to the depth and width of the furrow (figure 2*f*). In a PCA ordination summarizing this analysis, leaves that have essentially the same boundary curve but belong to different area groups plot very close to each other in morphological space, and the gradient between leaf area groups is less clear (figure 3*b*); both results are expected since leaf area was factored out in this analysis. However, although the boundaries between area groups is less clear, there remains a trend from leaves with a small area (lower right of the ordination) to leaves with a large area (left of the ordination), and there is minimal overlap between leaves with an area less than 8 cm^2^ and leaves with an area greater than 24 cm^2^ (figure 3*b*).

For our topological approach, we investigated the relationship between leaf area and leaf shape further by analysing how accurately leaf area can be inferred from just the two dominant principal components of shape derived from our topological representation of *Ginkgo* leaves. We estimated leaf area by performing a linear regression of leaf area over the first two principal components (the regression gradient points diagonally to the lower right of the PCA ordination (figure 5*a*)), and then compared this estimated area to the measured area of each leaf (figure 5*b*). We then quantified the discrepancy between predicted and measured areas by calculating an *R*^2^ statistic that shows 90% of the variation in the estimated leaf area is explained by variation in measured leaf area and *Ginkgo* leaf shape, as represented by our topological (PH) approach, is, therefore, strongly related to leaf area.

Taken together, our analyses of leaf shape versus leaf area suggest three things. Firstly, data points from different area groups overlap in PCA summaries of both our geometric and topological representation of *Ginkgo* leaves (figures 3*a* and 5*a*) and this highlights that while clearly an important mode of morphological variation, leaf area is not the only trait responsible for organizing the distribution of data points in *Ginkgo* leaf morphological space. This is emphasized by the offset between measured leaf areas and those predicted using our topological representation (figure 5*b*). Secondly, the weak leaf area trend that is present in our geometric scale-free analysis (figure 3*b*), suggests that leaf area may itself exert an influence on the other morphological traits such as furrow depth and the nature of the leaf margin. These observations may support the idea (based on work with angiosperm leaves rather than gymnosperm leaves) that leaf shape may be ‘a trait for which there are many quite varied functional trade-offs’ and that may be an ‘option that fine tunes the leaf to its conditions’ [11, p. 547]. Finally, our observations support reports indicating that leaf shape is at least correlated with leaf area in *Ginkgo* ([16,17], see also [46] for an area–shape correlation among bamboo leaves).

### 6.3. Image segmentation

Image segmentation—the partitioning of a digital image into multiple segments—is a key step in any study involving the computational analysis of digital imagery. In this study, the goal of image segmentation was to represent each leaf by its outline. For our sample of modern *Ginkgo* leaves, we were able to achieve segmentation computationally because the leaves themselves were whole, free from damage such as indentations in the leaf margin, and the images were free from major defects such as blurring. However, for the fossil *Ginkgo* leaves we have analysed, segmentation involved tracing the outline of each fossil leaf by hand rather than delineating the leaf margin computationally. In some cases of damage to a specimen, the original undamaged margin of a leaf was extremely faint, sometimes only visible using a microscope, whereas in others the leaf margin was interrupted by a scratch or hidden by a small piece of sediment. In situations such as these, knowledge of the processes leading to the formation and preservation of fossil leaves was used to calibrate a restoration of the fossil outline to what was judged to be its original state. This process introduces a source of potential error that is not quantified, and future work could explore how to automate elements of this image segmentation step, perhaps using a library of fossil leaf outlines produced by manual tracing to train a classifier, or perhaps repairing defects in the leaf margin computationally using techniques from inpainting (see [47]). The latter could be particularly valuable in studies of leaves where damage by insects is high such as in lowland moist tropical rainforests. Discussion of image segmentation is important because it can be a factor that limits the scope of studies that rely on the computational analysis of biological imagery. In particular, we feel that image segmentation will become a key issue if methods such as those we have described here are to be upscaled and automated to analyse large numbers of fossil leaves.

### 6.4. Future applications

The inclusion of fossil leaves in this exploratory analysis (figure 6) indicates that both the PH framework and geometric methods based on elastic curves have potential application to evolutionary and palaeoecological problems that require data on leaf shape in the geological past (e.g. [7,10,13–16]). Shape data derived from these approaches could also be used as classifiers in machine learning work to automate the classification of leaves in studies of modern and ancient plant diversity (cf. [48]), and could help quantify the nature and rate of leaf shape change during development (e.g. [25]) as well as investigate how leaf shape varies as a function of a tree's aspect.

For angiosperms, ‘leaf size and shape are selected by climate and are strongly correlated with climatic variables’ ([49], p. 266) and a clear next step is to apply our methods to angiosperm leaves in the context of climatic and palaeoclimatic analysis (e.g. [15]). In particular, given that our topological features measure the depth of indentations in the leaf margin, we are particularly interested to undertake quantitative analyses of angiosperm leaf margins. Such data could also feed into the climate leaf analysis multivariate programme (CLAMP) [50–52], which uses discrete categories to describe aspects of leaf form, and may enhance characters relating to the leaf margin (such as the regularity and closeness of leaf teeth) and the overall shape of leaves.

The methods we have described could also be used to quantify other planar shapes produced by plants such as the sepals, petals and tepals of flowers, which may enhance studies of the relationship between morphology and pollination biology (cf. [53]). As an illustration of the potential wider applicability of our methods, the long-shoot and short-shoot leaves of our modern *Ginkgo* leaves are well separated by our topological approach with minimal overlap between these two discrete classes (figure 5*c*). Given that long-shoot leaf morphology is thought to arise from the hydraulic limitation of long-shoot leaves during development [11], this highlights that our methods may be usefully applied to the problem of quantifying the relationship between morphology and the underlying physiological and developmental processes that are responsible for the generation of organic form.

## Data accessibility

The raw data for our manuscript consists of the images of the leaves we have studied (Mander *et al.* [54], Living and fossil Ginkgo leaves, Dryad, Dataset, https://doi.org/10.5061/dryad.7h44j0zsj). These images are available at: https://datadryad.org/stash/share/FMnz1duxMAX_RmH7gkCdq385OeWeUuTux9rinUzmSBo. Data and relevant code for this research work are stored in GitHub: https://github.com/h2metrics/h2metrics. https://github.com/haibinhang/TDA-of-ginkgo-leaves and have been archived within the Zenodo repository: https://doi.org/10.5281/zenodo.5590644, https://doi.org/10.5281/zenodo.5584877.
