# Supplementary material for: Geometric and topological approaches to shape variation in Ginkgo leaves
Source: R Soc Open Sci. 2021 Nov 24;8(11):210978. doi: 10.1098/rsos.210978 (PMC8611351; doi:10.1098/rsos.210978)
Supplement: Supplementary file 1 [file rsos210978_review_history.pdf]

THE ROYAL SOCIETY
PUBLISHING

ROYAL SOCIETY
OPEN SCIENCE

Geometric and topological approaches to shape variation in *Ginkgo* leaves

Haibin Hang, Martin Bauer, Washington Mio and Luke Mander

Article citation details
R. Soc. open sci. **8**: 210978.
http://dx.doi.org/10.1098/rsos.210978

Review timeline

Original submission:	18 June 2021
Revised submission:	22 October 2021
Final acceptance:	25 October 2021

Note: Reports are unedited and appear as submitted by the referee. The review history appears in chronological order.

Review History

RSOS-210978.R0 (Original submission)

Review form: Reviewer 1

Is the manuscript scientifically sound in its present form?
Yes

Are the interpretations and conclusions justified by the results?
Yes

Is the language acceptable?
Yes

Do you have any ethical concerns with this paper?
No

Have you any concerns about statistical analyses in this paper?
No

Recommendation?
Accept with minor revision (please list in comments)

Reports © 2021 The Reviewers; Decision Letters © 2021 The Reviewers and Editors; Responses © 2021 The Reviewers, Editors and Authors. Published by the Royal Society under the terms of the Creative Commons Attribution License http://creativecommons.org/licenses/by/4.0/, which permits unrestricted use, provided the original author and source are credited

Comments to the Author(s)
This paper is a well formulated study that proposes new tools/ methods to evaluate leaf shape, with G. biloba chosen as the target species to explore and test these tools/ methods. The methods and workflow are clearly described and the code for the analyse is publicly available allowing for others to use the methods.

I have a couple of minor requests, the colour choice in fig 3 should be revisited with a view to colour vision deficiency as red and green on the same plots should be avoided.

Line 74-77 leaf shape is linked to a number of different factors with a single reference made to a single review paper. I think it would be better if each factor had a separate citaion rather than linking all of these to the one study.

Review form: Reviewer 2

Is the manuscript scientifically sound in its present form?
Yes

Are the interpretations and conclusions justified by the results?
Yes

Is the language acceptable?
Yes

Do you have any ethical concerns with this paper?
No

Have you any concerns about statistical analyses in this paper?
No

Recommendation?
Accept with minor revision (please list in comments)

Comments to the Author(s)
The authors used two approaches (a geometric approach and a topological method) to study the variation and the corresponding determining factors for the leaf shape of ginkgo biloba. The fresh leaves and fossil leaves was used as materials. This manuscript was well prepared, and English writing is fluent. The methods appear to be appropriate. For me, using those methods to identify species might be problematic because many closely related species have very similar leaf shapes. However, at a single species level, those methods should be valid in distinguishing different cultivars or varieties. The current work belongs to the latter. Thus, I suggest accepting this manuscript after a minor revision.

Abstract
The current abstract has clearly summarized the background, material, methods and results. However, in the end of this section, the author had better use one or several sentences to further show the meanings or implications of this work in biology or ecology.

Lines 53 and 54: Arrange the key words in alphabet order.

Lines 68-71: The statements are clear and concise, but lack the support of references. I suggest authors to considering citing the following references:

Li, Y., Niklas, K.J., Gielis, J., Niinemets, U., Schrader, J., Wang, R., Shi, P. 2021. An elliptical blade is not a true ellipse, but a superellipse – Evidence from two Michelia species. Journal of Forestry Research, in press, https://doi.org/10.1007/s11676-021-01385-x

Schrader, J., Shi, P., Royer, D.L., Peppe, D.J., Gallagher, R.V., Li, Y., Wang, R., Wright, I.J. 2021. Leaf size estimation based on leaf length, width and shape. Annals of Botany, 128: 395−406

Shi, P., Liu, M., Ratkowsky, D.A., Gielis, J., Su, J., Yu, X., Wang, P., Zhang, L., Lin, Z., Schrader, J. 2019a. Leaf area-length allometry and its implications in leaf-shape evolution. Trees – Structure and Function 33: 1073−1085

Shi, P., Liu, M., Yu, X., Gielis, J., Ratkowsky, D.A. 2019b. Proportional relationship between leaf area and the product of leaf length width of four types of special leaf shapes. Forests 10: 178, https://doi.org/10.3390/f10020178

Shi, P., Yu, K., Niklas, K.J., Schrader, J., Song, Y., Zhu, R., Li, Y., Wei, H., Ratkowsky, D.A. 2021. A general model for describing the ovate leaf shape. Symmetry 2021, 13, 1524. https://doi.org/10.3390/sym13081524

Su, J., Niklas, K.J., Huang, W., Yu, X., Yang, Y., Shi, P. 2019. Lamina shape does not correlate with lamina surface area: An analysis based on the simplified Gielis equation. Global Ecology and Conservation 19: e00666, https://doi.org/10.1016/j.gecco.2019.e00666

Yu, X., Shi, P., Schrader, J., Niklas, K.J. 2020. Nondestructive estimation of leaf area for 15 species of vines with different leaf shapes. American Journal of Botany 107: 1481−1490

Line 112: I think, if possible, the following work also deserves mentioning:

Shi, P., Ratkowsky, D.A., Li, Y., Zhang, L., Lin, S., Gielis, J. 2018. General leaf-area geometric formula exists for plants – Evidence from the simplified Gielis equation. Forests 9: 714, https://doi.org/10.1016/10.3390/f9110714
Also see the above references, Li et al. (2021) and Shi et al. (2021).
You can say sth like: "Shi et al. (2018) found that the leaf shape of bamboo could be depicted by the simplified Gielis equation; Li et al. (2021) found that leaf shape of two Michelia species followed the superellipse equation; Shi et al. (2021) developed a general formula for describing ovate leaf shape in plants".

Line 173: Did this tree naturally grow on the campus or was it introduced from other places? Is there the age information or its DBH and height information? Is it a female tree or a male tree?

Lines 212-212: About the geodesic distance, it is better to show its mathematical expression. Maybe you can refer to Laga et al. (2014).

Line 230: Which were original factors (for obtaining the linearized factors) used for the PCA?

Line 517: I suggest the following references:

Lin, S., Niklas, K.J., Wan, Y., Hölscher, D., Hui, C., Ding, Y., Shi, P. 2020. Leaf shape influences the scaling of leaf dry mass vs. area: a test case using bamboos. Annals of Forest Science 77: 11

Shi, P., Ratkowsky, D.A., Li, Y., Zhang, L., Lin, S., Gielis, J. 2018. General leaf-area geometric formula exists for plants – Evidence from the simplified Gielis equation. Forests 9: 714

Decision letter (RSOS-210978.R0)

We hope you are keeping well at this difficult and unusual time. We continue to value your support of the journal in these challenging circumstances. If Royal Society Open Science can assist you at all, please don't hesitate to let us know at the email address below.

Dear Dr Mander

On behalf of the Editors, we are pleased to inform you that your Manuscript RSOS-210978 "Geometric and Topological Approaches to Shape Variation in Ginkgo Leaves" has been accepted for publication in Royal Society Open Science subject to minor revision in accordance with the referees' reports. Please find the referees' comments along with any feedback from the Editors below my signature.

We invite you to respond to the comments and revise your manuscript. Below the referees' and Editors' comments (where applicable) we provide additional requirements. Final acceptance of your manuscript is dependent on these requirements being met. We provide guidance below to help you prepare your revision.

Please submit your revised manuscript and required files (see below) no later than 7 days from today's (ie 04-Oct-2021) date. Note: the ScholarOne system will 'lock' if submission of the revision is attempted 7 or more days after the deadline. If you do not think you will be able to meet this deadline please contact the editorial office immediately.

Please note article processing charges apply to papers accepted for publication in Royal Society Open Science (https://royalsocietypublishing.org/rsos/charges). Charges will also apply to papers transferred to the journal from other Royal Society Publishing journals, as well as papers submitted as part of our collaboration with the Royal Society of Chemistry (https://royalsocietypublishing.org/rsos/chemistry). Fee waivers are available but must be requested when you submit your revision (https://royalsocietypublishing.org/rsos/waivers).

Thank you for submitting your manuscript to Royal Society Open Science and we look forward to receiving your revision. If you have any questions at all, please do not hesitate to get in touch.

Kind regards,
Royal Society Open Science Editorial Office
Royal Society Open Science
openscience@royalsociety.org

on behalf of Professor Brooke Flammang (Associate Editor) and Kevin Padian (Subject Editor)
openscience@royalsociety.org

Editor Comments to the Author:

Both reviewers find this manuscript to be well-written, one with experience with an earlier version who cites it as being greatly improved. Both reviewers offer minor edits that the authors should implement for final acceptance. We look forward to your revision, and thanks for submitting.

Reviewer comments to Author:
Reviewer: 1
Comments to the Author(s)
This paper is a well formulated study that proposes new tools/ methods to evaluate leaf shape, with G. biloba chosen as the target species to explore and test these tools/ methods. The methods and workflow are clearly described and the code for the analyse is publicly available allowing for others to use the methods.

I have a couple of minor requests, the colour choice in fig 3 should be revisited with a view to colour vision deficiency as red and green on the same plots should be avoided.

Line 74-77 leaf shape is linked to a number of different factors with a single reference made to a single review paper. I think it would be better if each factor had a separate citaion rather than linking all of these to the one study.

Reviewer: 2
Comments to the Author(s)
The authors used two approaches (a geometric approach and a topological method) to study the variation and the corresponding determining factors for the leaf shape of ginkgo biloba. The fresh leaves and fossil leaves was used as materials. This manuscript was well prepared, and English writing is fluent. The methods appear to be appropriate. For me, using those methods to identify species might be problematic because many closely related species have very similar leaf shapes. However, at a single species level, those methods should be valid in distinguishing different cultivars or varieties. The current work belongs to the latter. Thus, I suggest accepting this manuscript after a minor revision.

Abstract
The current abstract has clearly summarized the background, material, methods and results. However, in the end of this section, the author had better use one or several sentences to further show the meanings or implications of this work in biology or ecology.

Lines 53 and 54: Arrange the key words in alphabet order.

Lines 68-71: The statements are clear and concise, but lack the support of references. I suggest authors to considering citing the following references:

Li, Y., Niklas, K.J., Gielis, J., Niinemets, U., Schrader, J., Wang, R., Shi, P. 2021. An elliptical blade is not a true ellipse, but a superellipse – Evidence from two Michelia species. Journal of Forestry Research, in press, https://doi.org/10.1007/s11676-021-01385-x

Schrader, J., Shi, P., Royer, D.L., Peppe, D.J., Gallagher, R.V., Li, Y., Wang, R., Wright, I.J. 2021. Leaf size estimation based on leaf length, width and shape. Annals of Botany, 128: 395−406

Shi, P., Liu, M., Ratkowsky, D.A., Gielis, J., Su, J., Yu, X., Wang, P., Zhang, L., Lin, Z., Schrader, J. 2019a. Leaf area-length allometry and its implications in leaf-shape evolution. Trees – Structure and Function 33: 1073−1085

Shi, P., Liu, M., Yu, X., Gielis, J., Ratkowsky, D.A. 2019b. Proportional relationship between leaf area and the product of leaf length width of four types of special leaf shapes. Forests 10: 178, https://doi.org/10.3390/f10020178

Shi, P., Yu, K., Niklas, K.J., Schrader, J., Song, Y., Zhu, R., Li, Y., Wei, H., Ratkowsky, D.A. 2021. A general model for describing the ovate leaf shape. Symmetry 2021, 13, 1524. https://doi.org/10.3390/sym13081524

Su, J., Niklas, K.J., Huang, W., Yu, X., Yang, Y., Shi, P. 2019. Lamina shape does not correlate with lamina surface area: An analysis based on the simplified Gielis equation. Global Ecology and Conservation 19: e00666, https://doi.org/10.1016/j.gecco.2019.e00666

Yu, X., Shi, P., Schrader, J., Niklas, K.J. 2020. Nondestructive estimation of leaf area for 15 species of vines with different leaf shapes. American Journal of Botany 107: 1481−1490

Line 112: I think, if possible, the following work also deserves mentioning:

Shi, P., Ratkowsky, D.A., Li, Y., Zhang, L., Lin, S., Gielis, J. 2018. General leaf-area geometric formula exists for plants – Evidence from the simplified Gielis equation. Forests 9: 714, https://doi.org/10.1016/10.3390/f9110714
Also see the above references, Li et al. (2021) and Shi et al. (2021).
You can say sth like: "Shi et al. (2018) found that the leaf shape of bamboo could be depicted by the simplified Gielis equation; Li et al. (2021) found that leaf shape of two Michelia species followed the superellipse equation; Shi et al. (2021) developed a general formula for describing ovate leaf shape in plants".

Line 173: Did this tree naturally grow on the campus or was it introduced from other places? Is there the age information or its DBH and height information? Is it a female tree or a male tree?

Lines 212-212: About the geodesic distance, it is better to show its mathematical expression. Maybe you can refer to Laga et al. (2014).

Line 230: Which were original factors (for obtaining the linearized factors) used for the PCA?

Line 517: I suggest the following references:

Lin, S., Niklas, K.J., Wan, Y., Hölscher, D., Hui, C., Ding, Y., Shi, P. 2020. Leaf shape influences the scaling of leaf dry mass vs. area: a test case using bamboos. Annals of Forest Science 77: 11

Shi, P., Ratkowsky, D.A., Li, Y., Zhang, L., Lin, S., Gielis, J. 2018. General leaf-area geometric formula exists for plants – Evidence from the simplified Gielis equation. Forests 9: 714

===PREPARING YOUR MANUSCRIPT===

Your revised paper should include the changes requested by the referees and Editors of your manuscript. You should provide two versions of this manuscript and both versions must be provided in an editable format:
one version identifying all the changes that have been made (for instance, in coloured highlight, in bold text, or tracked changes);
a 'clean' version of the new manuscript that incorporates the changes made, but does not highlight them. This version will be used for typesetting.

Please ensure that any equations included in the paper are editable text and not embedded images.

Please ensure that you include an acknowledgements' section before your reference list/bibliography. This should acknowledge anyone who assisted with your work, but does not

qualify as an author per the guidelines at https://royalsociety.org/journals/ethics-policies/openness/.

While not essential, it will speed up the preparation of your manuscript proof if you format your references/bibliography in Vancouver style (please see https://royalsociety.org/journals/authors/author-guidelines/#formatting). You should include DOIs for as many of the references as possible.

If you have been asked to revise the written English in your submission as a condition of publication, you must do so, and you are expected to provide evidence that you have received language editing support. The journal would prefer that you use a professional language editing service and provide a certificate of editing, but a signed letter from a colleague who is a native speaker of English is acceptable. Note the journal has arranged a number of discounts for authors using professional language editing services (https://royalsociety.org/journals/authors/benefits/language-editing/).

===PREPARING YOUR REVISION IN SCHOLARONE===

To revise your manuscript, log into https://mc.manuscriptcentral.com/rsos and enter your Author Centre - this may be accessed by clicking on "Author" in the dark toolbar at the top of the page (just below the journal name). You will find your manuscript listed under "Manuscripts with Decisions". Under "Actions", click on "Create a Revision".

Attach your point-by-point response to referees and Editors at Step 1 'View and respond to decision letter'. This document should be uploaded in an editable file type (.doc or .docx are preferred). This is essential.

Please ensure that you include a summary of your paper at Step 2 'Type, Title, & Abstract'. This should be no more than 100 words to explain to a non-scientific audience the key findings of your research. This will be included in a weekly highlights email circulated by the Royal Society press office to national UK, international, and scientific news outlets to promote your work.

At Step 3 'File upload' you should include the following files:
-- Your revised manuscript in editable file format (.doc, .docx, or .tex preferred). You should upload two versions:
1) One version identifying all the changes that have been made (for instance, in coloured highlight, in bold text, or tracked changes);
2) A 'clean' version of the new manuscript that incorporates the changes made, but does not highlight them.
-- An individual file of each figure (EPS or print-quality PDF preferred [either format should be produced directly from original creation package], or original software format).
-- An editable file of each table (.doc, .docx, .xls, .xlsx, or .csv).
-- An editable file of all figure and table captions.
Note: you may upload the figure, table, and caption files in a single Zip folder.
-- Any electronic supplementary material (ESM).
-- If you are requesting a discretionary waiver for the article processing charge, the waiver form must be included at this step.
-- If you are providing image files for potential cover images, please upload these at this step, and inform the editorial office you have done so. You must hold the copyright to any image provided.
-- A copy of your point-by-point response to referees and Editors. This will expedite the preparation of your proof.

At Step 6 'Details & comments', you should review and respond to the queries on the electronic submission form. In particular, we would ask that you do the following:
-- Ensure that your data access statement meets the requirements at https://royalsociety.org/journals/authors/author-guidelines/#data. You should ensure that you cite the dataset in your reference list. If you have deposited data etc in the Dryad repository, please only include the 'For publication' link at this stage. You should remove the 'For review' link.
-- If you are requesting an article processing charge waiver, you must select the relevant waiver option (if requesting a discretionary waiver, the form should have been uploaded at Step 3 'File upload' above).
-- If you have uploaded ESM files, please ensure you follow the guidance at https://royalsociety.org/journals/authors/author-guidelines/#supplementary-material to include a suitable title and informative caption. An example of appropriate titling and captioning may be found at https://figshare.com/articles/Table_S2_from_Is_there_a_trade-off_between_peak_performance_and_performance_breadth_across_temperatures_for_aerobic_scope_in_teleost_fishes_/3843624.

At Step 7 'Review & submit', you must view the PDF proof of the manuscript before you will be able to submit the revision. Note: if any parts of the electronic submission form have not been completed, these will be noted by red message boxes.

Author's Response to Decision Letter for (RSOS-210978.R0)

See Appendix A.

Decision letter (RSOS-210978.R1)

We hope you are keeping well at this difficult and unusual time. We continue to value your support of the journal in these challenging circumstances. If Royal Society Open Science can assist you at all, please don't hesitate to let us know at the email address below.

Dear Dr Mander,

I am pleased to inform you that your manuscript entitled "Geometric and Topological Approaches to Shape Variation in Ginkgo Leaves" is now accepted for publication in Royal Society Open Science.

If you have not already done so, please ensure that you send to the editorial office an editable version of your accepted manuscript, and individual files for each figure and table included in your manuscript. You can send these in a zip folder if more convenient. Failure to provide these files may delay the processing of your proof.

Please remember to make any data sets or code libraries 'live' prior to publication, and update any links as needed when you receive a proof to check - for instance, from a private 'for review' URL to a publicly accessible 'for publication' URL. It is good practice to also add data sets, code and other digital materials to your reference list.

Our payments team will be in touch shortly if you are required to pay a fee for the publication of the paper (if you have any queries regarding fees, please see https://royalsocietypublishing.org/rsos/charges or contact authorfees@royalsociety.org).

The proof of your paper will be available for review using the Royal Society online proofing system and you will receive details of how to access this in the near future from our production office (openscience_proofs@royalsociety.org). We aim to maintain rapid times to publication after acceptance of your manuscript and we would ask you to please contact both the production office and editorial office if you are likely to be away from e-mail contact to minimise delays to publication. If you are going to be away, please nominate a co-author (if available) to manage the proofing process, and ensure they are copied into your email to the journal.

Please see the Royal Society Publishing guidance on how you may share your accepted author manuscript at https://royalsociety.org/journals/ethics-policies/media-embargo/. After publication, some additional ways to effectively promote your article can also be found here https://royalsociety.org/blog/2020/07/promoting-your-latest-paper-and-tracking-your-results/.

On behalf of the Editors of Royal Society Open Science, thank you for your support of the journal and we look forward to your continued contributions to Royal Society Open Science.

Kind regards,
Royal Society Open Science Editorial Office
Royal Society Open Science
openscience@royalsociety.org

on behalf of Professor Brooke Flammang (Associate Editor) and Kevin Padian (Subject Editor)
openscience@royalsociety.org

Follow Royal Society Publishing on Twitter: @RSocPublishing
Follow Royal Society Publishing on Facebook:
https://www.facebook.com/RoyalSocietyPublishing.FanPage/
Read Royal Society Publishing's blog:
https://royalsociety.org/blog/blogsearchpage/?category=Publishing

Appendix A

School of Environment, Earth and Ecosystem Sciences
The Open University, Walton Hall
Milton Keynes, MK7 6AA, UK
Tel: +44 (0) 7920 844184
Email: luke.mander@gmail.com

11th October 2021

Dear Editor,

Please find attached a revised manuscript entitled "Geometric and Topological Approaches to Shape Variation in *Ginkgo* Leaves" (authors: Hang*, Bauer*, Mio and Mander**,) [*denotes equal contribution to this work, **corresponding author]. We thank the reviewers for their comments and detail our responses below.

Reviewer 1
Figure 3. We have updated Figure 3 with an alternative colour palette.

L74–77 references. We have added the following five references to this section:

Karban R, Thaler JS. 1999. Plant phase change and resistance to herbivory. *Ecology* 80: 510–517.
Niklas KJ, Cobb ED, Spatz H-C. 2009. Predicting the allometry of leaf surface area and dry mass. *American Journal of Botany* 96: 531–536.
Sack L, Cowan PD, Jaikumar N, Holbrook NM. 2003. The 'hydrology' of leaves: co-ordination of structure and function in temperate woodybambooba species. *Plant, Cell & Environment* 26: 1343–1356.
Vogel S. 1970. Convective cooling at low airspeeds and the shapes of broad leaves. *Journal of Experimental Botany* 21: 91–101.
Yano S, Terashima I. 2004. Developmental process of sun and shade leaves in *Chenopodium album* L. *Plant, Cell & Environment* 27: 781–793.

Reviewer 2
Abstract. Abstract edited to include a line on possible applications for this work.
Key word order. We have elected to keep these ordered by their significance to the research.
L 173. Details on the specimen *Ginkgo* tree added.
Line 212. For the metrics that we use in this analysis, there is no explicit formula for the geodesic distance, and it has to be found by solving an optimization problem. For closed curves the geodesic distance in Laga et al. (2014) also doesn't have an explicit formula. Consequently, we have left L212 unchanged.
References (L68–51 and L517). We have added the following text and three of the suggested references to our manuscript:

Additionally, Shi *et al.* (2018) found that the leaf shape of bamboo could be depicted by the simplified Gielis equation, while Li *et al.* (2021) noted that leaf shape of two *Michelia* species followed the superellipse equation, and Shi *et al.* (2021) developed a general formula for describing ovate leaf shape in plants.

Li Y, Niklas KJ, Gielis J, Niinemets U, Schrader J, Wang R, Shi P. 2021. An elliptical blade is not a true ellipse, but a superellipse – Evidence from two *Michelia* species. *Journal of Forestry Research* in press.

Shi P, Ratkowsky DA, Li Y, Zhang L, Lin S, Gielis J. 2018. General leaf-area geometric formula exists for plants – Evidence from the simplified Gielis equation. *Forests* 9: 714.
Shi P, Yu K, Niklas KJ, Schrader J, Song Y, Zhu R, Li Y, Wei H, Ratkowsky DA. 2021. A general model for describing the ovate leaf shape. *Symmetry* 13: 1524.

Authors own changes
We have updated the github link for our topological approach: https://github.com/haibinhang/TDA-of-ginkgo-leaves

Yours Faithfully,

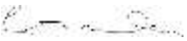

Dr Luke Mander
